# Gold Nanoparticles Prepared with *Phyllanthus emblica* Fruit Extract and *Bifidobacterium animalis* subsp. *lactis* Can Induce Apoptosis via Mitochondrial Impairment with Inhibition of Autophagy in the Human Gastric Carcinoma Cell Line AGS

**DOI:** 10.3390/nano11051260

**Published:** 2021-05-11

**Authors:** Rongbo Wang, Xingyue Xu, Aditi Mitra Puja, Haribalan Perumalsamy, Sri Renukadevi Balusamy, Hoon Kim, Yeon-Ju Kim

**Affiliations:** 1 Graduate School of Biotechnology, College of Life Science, Kyung Hee University, Yongin-si 17104, Korea; wangrongbo@khu.ac.kr (R.W.); xingyue5125@khu.ac.kr (X.X.); aditi@khu.ac.kr (A.M.P.); harijai2004@gmail.com (H.P.); 2 Department of Food Science and Biotechnology, Sejong University, Gwangjin-gu, Seoul 05006, Korea; renucoimbatore@gmail.com; 3 Department of Oriental Medicine Biotechnology, Kyung Hee University, Yongin-si 17104, Korea

**Keywords:** *Phyllanthus emblica* fruit, *Bifidobacterium animalis* subsp. *lactis*, gold nanoparticles, gastric cancer, apoptosis, autophagy

## Abstract

(1) Background: Nanotechnology is being widely applied for anticancer strategies with few side effects. Nanoparticles (NPs) prepared from natural extracts are promising candidates for cancer treatment because of their unique physicochemical characteristics. This study aimed to prepare gold nanoparticles (AuNPs) from *Phyllanthus emblica* fruit extract (PEFE) using *Bifidobacterium animalis* subsp. *lactis* (*B. lactis*) and to evaluate their anticancer activity against the human gastric adenocarcinoma cell-line (AGS). (2) Methods: The safety of microbial biosynthesis AuNPs (PEFE-AuNPs) was assessed by evaluating the cytotoxicity. The anticancer activity of PEFE-AuNPs was investigated in AGS cells in terms of apoptosis and autophagy. (3) Results: PEFE-AuNPs exhibited significant cytotoxicity against AGS cells but not against normal cells. The apoptosis induced by PEFE-AuNPs in AGS cells was associated with PTEN-induced kinase 1 (PINK1)-Parkin mediated reduction of mitochondrial membrane potential and activation of intracellular signaling apoptosis pathways. The anticancer activity of PEFE-AuNPs was associated with induction of apoptosis through inhibition of autophagy, downregulation of LC3-II/LC3-I and Beclin-1 expression, and upregulation of p62 expression in AGS cells. (4) Conclusions: This study is the first to demonstrate the anticancer activity of PEFE-AuNPs against AGS cells. Our results provide a good starting point for the development of new anticancer products based on gold nanoparticles of *P. emblica* fruit extract.

## 1. Introduction

Gastric cancer (GC) is one of the most common cancers and has the second highest cancer-related mortality rate in the world [1,2]. Although the incidence of GC has a large geographic variation, there is a consistently high incidence of 60% in East Asia [2,3]. Despite the many effective treatment modalities for GC, such as surgical excision, chemotherapy, radiotherapy, targeted therapy, and a combination of these interventions, GC generally remains to have a poor prognosis and low survival rates [4]. Therefore, developing novel and highly efficient therapeutic strategies for GC is strongly recommended [5].

In the past few decades, nanoparticles (NPs), which are particles typically with a diameter of 1–100 nm, have been broadly applied in various fields [6]. In particular, NPs have received considerable attention in cancer research, as they are able to destroy tumor cells without damaging normal cells, resulting in an improved safety profile compared to conventional drugs [7,8]. Among the variety of metallic NPs that have been developed and evaluated for their characteristics [9], gold nanoparticles (AuNPs) have gained the most attention in the fields of diagnostics, therapeutics, pharmacology, and immunology [10,11]. AuNPs have been utilized in medical and therapeutic applications because of their unique properties, including their synthesis and functionalization compatibility, low toxicity, and detection capabilities [9,12]. Generally, physical, chemical, and biological methods are available for the synthesis of NPs. NPs synthesized by physical and chemical methods are generally regarded as expensive, toxic, and hazardous, which are known to create serious environmental problems [13]. However, the biological methods, which involve plant tissues and/or microorganisms, are favored, as they are relatively simple, rapid, eco-friendly, and able to produce stable final products compared to the chemical methods [14]. In particular, processes using bacterial cells have been widely preferred for preparing AuNPs, because they are more cost-effective and eco-friendly than synthetic methods [15,16]. In addition, there is no need to add any external stabilizing agents for biosynthesis of AuNPs, because biological ingredients present in plants and microorganisms themselves can be responsible for reducing, stabilizing, and capping agents [14]. AuNPs have been widely applied in numerous fields, such as diagnostic, bioimaging, catalytic, bioassay, sensing, drug delivery, cancer therapy, and antimicrobial [14]. In particular, AuNPs can exert increased anticancer potentials compared with the therapeutic effects they contain. This is owing to more specific targeting to tumor tissues via improved pharmacokinetics and pharmacodynamics, and active intracellular delivery [17].

*Phyllanthus emblica* L., also known as Indian gooseberry or amla, belongs to the family Phyllanthaceae. Its fruit has been used in the preparation of herbal medicines, such as those associated with Ayurveda, a traditional alternative medicine system with historical roots in India [18]. *P. emblica* is known to have high amounts of vitamin C and polyphenols [19] and exhibits a variety of physiological activities, such as antioxidant [20], anticancer [21], anti-inflammatory [22], antibacterial [23], anti-obesity [24], anti-diabetes [25], and hepatoprotective [26] properties. In particular, hydrolysable tannin-derived compounds, such as gallic acid, ellagic acid, chebulagic acid, corilagin, and pyrogallol, in addition to flavonoid quercetin were reported that they can be responsible for the anticancer activity in the *P. emblica* [21]. However, the anticancer activity of AuNPs prepared with *P. emblica* extract has currently not been investigated. Therefore, in this study, we aimed to prepare AuNPs using the extract of *P. emblica* fruit via microbial biosynthesis and to investigate the anticancer activity of these AuNPs against GC. In addition, we explored the mechanisms underlying the anticancer activity of the AuNPs, particularly their involvement in apoptosis and autophagy.

## 2. Materials and Methods

### 2.1. Reagents

Cell culture reagents such as Dulbecco’s modified Eagle’s medium (DMEM), Roswell Park Memorial Institute (RPMI) 1640 medium, penicillin-streptomycin (PS), and fetal bovine serum (FBS) were purchased from GenDEPOT (Katy, TX, USA). Dimethyl sulfoxide (DMSO), 3-(4,5-dimethylthiazol-2-yl)-2,5-diphenyltetrazolium bromide (MTT), Hoechst 33258, gold (III) chloride trihydrate (HAuCl_4_·3H_2_O) and hydrochloric acid were obtained from Sigma-Aldrich (St. Louis, MO, USA). Propidium iodide (PI) was obtained from Invitrogen (Carlsbad, CA, USA). All primers were designed by Macrogen (Seoul, Republic of Korea). Antibodies were purchased from Abcam (Cambridge, UK) and Cell Signaling Technology, Inc. (Danvers, MA, USA). L-cysteine hydrochloride monohydrate was purchased from Samchun Chemicals (Seoul, Korea).

### 2.2. Biosynthesis of PEFE-AuNPs

The plants in this research were carried out in accordance with guidelines provided by Kyung Hee University, Yongin-si, Gyeonggi-do, Republic of Korea. *Phyllanthus emblica* fruits (*P. emblica*) were purchased from the market at Coimbatore (Tamil Nadu, India). The fruits were authenticated by Professor P. Jayaraman, with specimen voucher number PARC/2017/3907 and stored at the Plant Anatomy Research Centre, Chennai, Tamil Nadu, India. The dried *P. emblica* fruits were grounded and macerated with 70% ethanol at room temperature. After that, the solution was concentrated using a rotary evaporator at 42 °C to obtain the *P. emblica* fruit extract (PEFE). The strain was isolated from yogurt, the similarity of 16S rRNA sequences was 99% to *Bifidobacterium animalis* subsp. *lactis* (*B. lactis*) deposited in NCBI GenBank under accession number CP001606, and was cultured in MRS (de Man, Rogosa and Sharpe) broth (Becton, Dickinson and Company, Franklin Lakes, NJ, USA). The bacterial cells were harvested by centrifugation at 3000 rpm for 10 min. The method of AuNP biosynthesis was based on a previous report [27], with slight modifications. Briefly, the collected cells were washed with distilled water several times and suspended in 10 mL of phosphate buffer (50 mM, pH 7.0). For the one-pot synthesis, 2 mM HAuCl_4_·3H_2_O and 0.08 mg/mL of PEFE were added to the cell suspension, followed by incubation at 37 °C for 24 h. The mixture was sonicated in ice water for 60 min and centrifuged at 3000 rpm for 10 min. The precipitated pellets (PEFE-AuNPs) were collected by centrifugation (12,000 rpm, 10 min, 4 °C), washed exhaustively, and resuspended in sterile water. The suspension was air-dried to obtain the powdered PEFE-AuNPs.

### 2.3. Physicochemical Characterization of PEFE-AuNPs

The absorption spectrum of PEFE-AuNPs was obtained using a Cary 60 ultraviolet-visible (UV-Vis) spectrophotometer (Agilent Technologies, Inc., Santa Clara, CA, USA) at 300–800 nm. A JEM-2100F field emission-transmission electron microscope (FE-TEM; JEOL, Ltd., Tokyo, Japan) was used to evaluate the size and morphology of PEFE-AuNPs at 200 kV. Images were obtained by suspending a drop of the purged molecule on a TEM network. X-ray diffraction (XRD), energy-dispersive X-ray (EDX) spectrum analysis and Fourier transform infrared spectroscopy (FT-IR) were used to monitor pellet formation, and selected area electron diffraction (SAED) was used to determine the crystalline structure of the NPs. The volume of the polymer grafted to the surface of the PEFE-AuNPs and the thermal stability of PEFE-AuNPs were evaluated by thermos gravimetric analysis (TGA) at temperatures of 30–600 °C. The dynamic light scattering (DLS) particle analyzer (Otsuka Electronics, Shiga, Japan) was used to determine the size and dispersal nature of the nanoparticles.

### 2.4. Hemocompatibility Assay

Defibrinated sheep blood was obtained from Kisan Bio Co., Ltd. (Seoul, Korea). Red blood cells were isolated by washing whole blood with PBS three times, followed by centrifugation (3000 rpm, 5 min). To evaluate the hemocompatibility of PEFE-AuNPs, the NP sample containing PEFE-AuNPs was diluted in 1 mL of PBS to a concentration of 150 μg/mL, and the sample solution was incubated with the red blood cell suspension at a ratio of 1:100 (*v*/*v*). The mixture was centrifuged at 13,000 rpm for 10 min to obtain the cell-free supernatant. Finally, the absorbance of the supernatant was measured at 545 nm using a SpectraMax^®^ ABS Microplate Reader (Molecular Devices, LLC., San Jose, CA, USA), and the hemocompatibility of the sample was evaluated against the negative control, which contained PBS and red blood cells only.

### 2.5. Evaluation of Intracellular Uptake and Localization of PEFE-AuNPs

To visualize the uptake capacity of PEFE-AuNPs, the human gastric carcinoma cell line AGS was obtained from Korean Cell Line Bank (KCLB, Seoul, Korea), and AGS cells were observed at 5 min and 3 h after treatment with PEFE-AuNPs. To monitor the distribution of the NPs within the cells, bright-field microscopy (Olympus Optical Co., Ltd., Tokyo, Japan) and enhanced dark-field (EDF) microscopy (CytoViva Inc., Auburn, AL, USA) were subsequently used to obtain the photographic results.

### 2.6. Cell Viability Assay

Two types of normal cell lines, the human epidermal keratinocyte cell line HaCaT and human dermal fibroblast cell line NHDF, were purchased from KCLB (Seoul, Korea). HaCaT and NHDF cells were cultured in DMEM containing 10% FBS and 1% PS, and AGS cells were cultured in RPMI-1640 medium containing 10% FBS and 1% PS. To assess the cytotoxic effect of PEFE-AuNPs, each cell suspension (1 × 10^4^ cells/well) was seeded in a 96-well plate. After stabilization for 24 h, the culture supernatant was removed by suction, and fresh media containing PEFE-AuNPs at concentrations of 50, 80, and 100 μg/mL were added to the cells. MTT assay was carried out 6 h after treatment according to a previously reported method [28].

### 2.7. Colony Formation Analysis by Trypan Blue Staining

AGS cells were seeded onto a cell culture dish (35 × 10 mm), treated with the NP samples at concentrations of 80 and 100 μg/mL for 24 h, and rinsed with PBS three times. To visualize the colony-forming cells, AGS cells were stained with tryphan blue solution (Thermo Fisher Scientific, Waltham, MA, USA) for 5 min, observed using optical microscopy, and analyzed using the Image J software (US National Institute of Health, Bethesda, MD, USA).

### 2.8. Hoechst 33,258 and Propidium Iodide (PI) Staining

AGS cells treated with the NP samples were stained with Hoechst 33,258 or PI to observe the morphology of the cells and to quantify the number of apoptotic cells. Briefly, AGS cells (1 × 10^5^ cells) were seeded onto a cell culture dish, stabilized for 48 h, and subsequently treated with the NP samples for 24 h. After staining with Hoechst 33,258 or PI, the cells were observed using a Leica DM IRB fluorescence microscope (Leica Microsystems, Wetzlar, Germany).

### 2.9. Reactive Oxygen Species (ROS) and Mito-SOX Quantification

By using Mito-Tracker^®^ Green (MTG) FM (Thermo Fisher Scientific, Waltham, MA, USA) the mitochondria were stained. The fluorescent color that held inside the mitochondria of cells, separately. After treatment with our samples, the AGS cells (1 × 10^5^ cells) were incubated with MitoTracker^®^ Green (0.1 μM) for 1 h at 37 °C and then washed three times with PBS. Cells were observed by the microscope (Leica Microsystems, Wetzlar, Germany). Intracellular ROS release was detected according to a Cellular ROS/Superoxide Detection Assay Kit (Abcam, Cambridge, MA, USA). AGS cells (1 × 10^5^ cells) were grown in 6-well plates and treated respectively with different concentrations of PEFE and PEFE-AuNPs for 24 h. Then, 0.4 μL of oxidative stress detection and superoxide detection reagents were added. Following an incubation period of 30 min, fluorescence was measured using microscope (Leica Microsystems, Wetzlar, Germany).

### 2.10. Cytosolic-Associated Protein Light Chain 3 (LC3) Immunofluorescence Staining

AGS cells (1 × 10^5^ cells) were grown in 6-well plates and treated respectively with different concentrations of PEFE and PEFE-AuNPs for 24 h. Cells were fixed by 4% paraformaldehyde and permeabilization using 0.1% Triton X-100 for 20 min. Cells washed with PBS, then blocked with 2% BSA in PBS for 1 h. The antibody of LC3 was incubated for 3 h at room temperature followed by a 30 min incubation with fluorescein isothiocyanate (FITC)-conjugated secondary antibody. After washing in PBS, the fluorescence was snapped using a Leica fluorescence microscope (Leica Microsystems, Wetzlar, Germany) and quantified using Image J software.

### 2.11. Quantitative Real-Time PCR (qRT-PCR)

The mRNA expression was evaluated using qRT-PCR. Briefly, total RNA was extracted using TRIzol reagent (Bioline, Brisbane, Australia), and mRNA was reverse transcribed to cDNA using the First-Strand Synthesis Kit (Invitrogen, Carlsbad, CA, USA). qRT-PCR was performed using amfiSure qGreen Q-PCR Master Mix (GenDEPOT, Katy, TX, USA) and a Rotor-Gene qRT-PCR machine (QIAGEN, Seoul, Korea). The primer sequences used are listed in Supplementary Table S1. The relative expression of the target genes against the endogenous control (β-actin) was calculated using the 2−ΔΔCt method (ΔΔCt = ΔCt[treated] − ΔCt[control]).

### 2.12. Quantitative Real-Time PCR (qRT-PCR)

Total protein was obtained from NP-treated AGS cells using RIPA (Radio-Immunoprecipitation Assay) lysis buffer (Thermo Fisher Scientific, Rockford, IL, USA) containing a protease inhibitor cocktail (GenDEPOT, Katy, TX, USA). Equal amounts (50 µg) of total protein were separated by sodium dodecyl sulfate-polyacrylamide gel (SDS-PAGE) containing 10% SDS and transferred onto a polyvinylidene fluoride membrane (PVDF; Thermo Fisher Scientific, Waltham, MA, USA) using the Protein Gel Electrophoresis Chamber System (Thermo Fisher Scientific, Rockford, IL, USA). Blots were blocked with PBS containing 5% skimmed milk for 2 h at room temperature and incubated with primary antibodies (Bax, Bcl-2, cytochrome c, caspase 9, caspase 3, LC3-I/II, Beclin-1, p62, PTEN-induced kinase 1 (PINK1), Parkin, TOM20, and β-actin) overnight at 4 °C. All primary antibodies were purchased from Thermo Fisher Scientific (Waltham, MA, USA). After incubation, the membrane was washed with PBS and incubated with horse radish peroxidase (HRP)-conjugated secondary antibody solution (Thermo Fisher Scientific, Waltham, MA, USA) for 1 h. Protein bands were visualized with the Enhanced Chemiluminescence (ECL) reagent (Thermo Fisher Scientific, Waltham, MA, USA) and quantified using the Image J software.

### 2.13. Statistical Analysis

The results were expressed as the mean ± standard deviation (SD) of three independent experiments, and the differences were evaluated by Student’s T-test or one-way analysis of variance (ANOVA). All differences were considered statistically significant between untreated control and tested sample at *p* < 0.05, *p* < 0.01, and *p* < 0.001.

## 3. Results and Discussion

### 3.1. Biosynthesis and Physiochemical Characterization of PEFE-AuNPs

**Figure 1:**
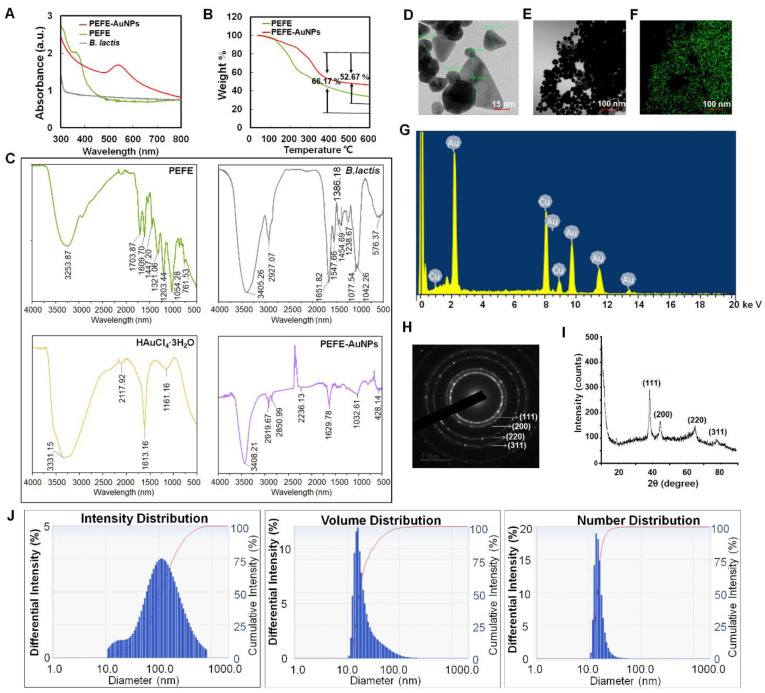
Physiochemical characterization of nanoparticle samples. (**A**) UV–Vis spectrum. (**B**) Thermos gravimetric analysis (TGA) spectrum. (**C**) Fourier-transform infrared (FT-IR) spectrum. (**D**) Field emission transmission electron microscope (FE-TEM) image of PEFE-AuNPs. (**E**,**F**) Elemental mapping analysis of PEFE-AuNPs, (**G**) Energy-dispersive X-ray (EDX) analysis of PEFE-AuNPs, (**H**) Selected area electron diffraction (SAED) image of PEFE-AuNPs, (**I**) X-ray diffraction (XRD) spectrum of PEFE-AuNPs, (**J**) dynamic light scattering (DLS) spectrum of PEFE-AuNPs.

The synthesis of AuNPs can be easily evaluated by observing the change in color of the culture medium from yellow to deep purple [14]. By observing such a color change, the synthesis of PEFE-AuNPs was confirmed (Figure 1A). Although there was no plasmonic absorbance observed for either the *B. lactis* sample or PEFE alone, the λmax of PEFE-AuNPs was observed at a wavelength of 545 nm, demonstrating the successful synthesis of AuNPs (Figure 1A). As shown in Supplementary Figure S1, we established the optimized conditions for the synthesis of PEFE-AuNPs in terms of PEFE and HAuCl_4_·3H_2_O concentrations, incubation time, and reaction pH. The optimized conditions included the following: 0.08 mg/mL of PEFE solution, 2 mM HAuCl_4_·3H_2_O, 24 h incubation period, and pH 7.0. Using TGA to determine the thermal stability of the polymers, lower weight loss was observed in PEFE-AuNPs (52.67%) than in PEFE (66.17%), indicating that PEFE-AuNPs have a more favorable thermal stability than PEFE (Figure 1B). On the other hand, stability test depending on the storage period was examined and provided in Supplementary Figure S2. The result reveals that PEFE-AuNPs were quite stable for 30 days after synthesis, but drastically degraded after 90 days. To identify the probable functional groups of PEFE-AuNPs, the FT-IR spectra of the samples were analyzed using the online database for natural substances and the in-house FT-IR spectral library. As shown in Figure 1C, the PEFE-AuNPs exhibited similar absorption patterns or little shift from the other three samples. The major stretching appearing at 3000–3500 cm^−1^ indicates the presence of O-H stretch which signifies the presence of phenols and flavonoids [29]. A little shifting occurs here, suggesting that the carbonyl group in the PEFE extract and *B. lactis* capped and stabilized [30]. The bands observed at 2919.67 and 2850.99 cm^−1^ in PEFE-AuNPs and 2927.70 cm^−1^ in *B. lactis* are due to the C-H bonds from the methyl or methylene groups [31]. The signals from 1703.87 to 1609.32 cm^−1^ of PEFE correspond to the aromatic double bonds (C=C) and carbonyl groups (C=O) [32]. The band at 1032.6 cm^−1^ of PEFE-AuNPs confirms the presence of the aliphatic ether (C-O) where shifting occurs from the peak at 1203.44 cm^−1^ of PEFE after capping with Au-NPs [33]. Taken together, the results suggest that PEFE-AuNPs was composed of a mixed structure comprising a specific structure of individual *B. lactis*, HAuCl_4_·3H_2_O, and PEFE.

As shown in Figure 1D,E, FE-TEM images showed that PEFE-AuNPs were 5–60 nm in size and formed circular, triangular, and polygonal nanohybrids [34]. Through elemental mapping analysis, the spatial distribution of elements demonstrated the spread of AuNPs (Figure 1F). The density of Au was noticeably increased, indicating that Au was the main element in the synthesized AuNPs. In the EDX spectrum analysis, the highest peak of optical absorption at 2.1 keV corresponded to Au (Figure 1G), and the abundant metal group at 8 keV was confirmed as a copper grid. The SAED image showed the diffraction, metallic characteristics, and circular structure of PEFE-AuNPs, suggesting the formation of crystalline NPs (Figure 1H). The XRD analysis also provides information on the space group, the crystallographic system, or the size of the crystallites. The peak pattern in the XRD image shows four peaks indexed to (111), (200), (220), and (311), and the highest peak corresponds to the (111) orientation (Figure 1I), indicating the gold lattice planes of Bragg’s reflection [35]. In addition, four diffraction peaks, 38.333°, 44.384°, 64.917°, and 77.537°, at the 2θ values were identified. Taken together, we successfully synthesized PEFE-AuNPs with PEFE and Au ions through an eco-friendly microbial biosynthetic process using an enzymatic system of *B. lactis*. The particle size distribution was analyzed by DLS analysis. Unlike TEM, DLS also takes the organic shell into account determining the whole size of the conjugates in the colloids or their average hydrodynamic size. Due to the number of biomolecules covering each NP in colloids is a big amount, the hydrodynamic size obtained by DLS measurement is considerably greater than the size obtained by TEM analysis [36]. The size range obtained by DLS analysis shows that the PEFE-AuNPs have a non-uniform distribution, with an average particle size of 133.4 nm (Figure 1J).

### 3.2. Cytotoxic Effect of PEFE-AuNPs against Normal and Cancer Cells

**Figure 2:**
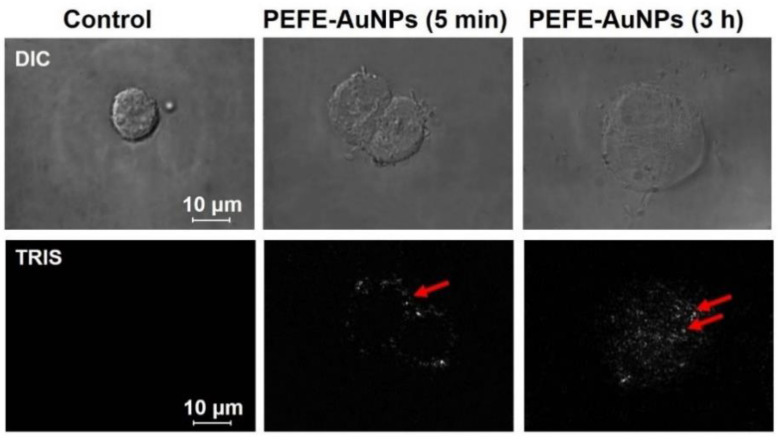
Enhanced dark-field (EDF) microscopic images of PEFE-AuNPs-treated AGS cells. The bright white spots inside the AGS cells represent the aggregation and location of PEFE-AuNPs. DIC, differential interference contrast; TIRS, thermal infrared sensor.

To evaluate the cellular uptake of PEFE-AuNPs, AGS cells were observed using EDF microscopy after PEFE-AuNPs treatment (Figure 2). The results showed that PEFE-AuNPs rarely accumulated inside the cells 5 min after the induction. However, after 3 h, the increased brightness indicated a drastic increase in the uptake of PEFE-AuNPs, suggesting that the synthesized PEFE-AuNPs can be absorbed by cells.

**Figure 3:**
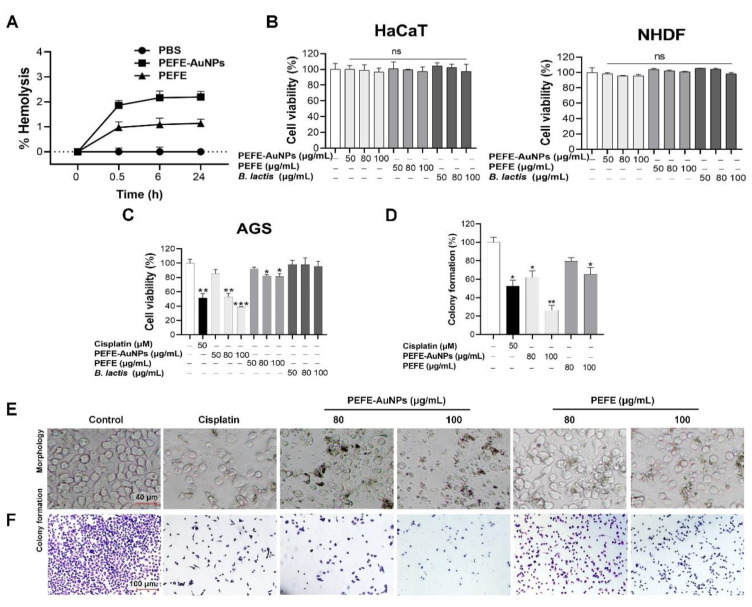
Cytotoxic effect of PEFE-AuNPs against normal and cancer cells. (**A**) The hemocompatibility of PEFE-Au NPs and PEFE. (**B**,**C**) The cytotoxic effect of PEFE-AuNPs, PEFE, and *B. lactis* against normal (HaCaT and NHDF) and cancer (AGS) cells. The cisplatin was used as positive control. (**D**–**F**) Microscopic images and colony formation of AGS cells treated with PEFE-AuNPs and PEFE. The cisplatin was used as positive control. *, *p* < 0.05, **, *p* < 0.01, ***, *p* < 0.001.

The sensitivity of mammalian cells to NPs and the biosafety concentrations of such biomaterials must be determined before any therapeutic trial or other potential biomedical applications [37,38]. Hemocompatibility is an essential requirement in evaluating the safety of biomaterials including NPs [30]. Thus, we determined the hemocompatibility of PEFE-AuNPs using red blood cells isolated from sheep blood. As shown in Figure 3A, PEFE-AuNPs induced hemolysis (2%) more than PEFE (1%) within 24 h, suggesting that PEFE-AuNPs are little more cytotoxic than PEFE. As hemolysis of less than 5% is considered safe for humans [39], PEFE-AuNPs did not compromise hemocompatibility.

The cytotoxic effects of PEFE-AuNPs, PEFE, and *B. lactis* were evaluated using two types of normal human cells, HaCaT cells (keratinocytes) and NHDF cells (fibroblasts). As shown in Figure 3B, no statistically significant differences were observed in terms of cytotoxicity between the control group (untreated cells) and the treatment groups (cells treated with *B. lactis*, PEFE, or PEFE-AuNPs at concentrations of 50–100 μg/mL), indicating that all samples were not toxic to the normal cells at the indicated concentrations. In contrast, PEFE-AuNPs significantly decreased the cell viability in a dose-dependent manner compared to PEFE and *B. lactis*. The half-maximal inhibitory concentration (IC50) of PEFE-AuNPs was approximately 80 µg/mL. Thus, 80 and 100 µg/mL were used in the subsequent evaluation of the anticancer activity of PEFE-AuNPs and PEFE against AGS cells (Figure 3C). The cell morphology and colony formation of AGS cells determined by microscopic observation are illustrated in Figure 3D–F. The results showed that the number of AGS cell colonies was higher in the control group than in the groups treated with PEFE-AuNPs or PEFE at concentrations of 80 and 100 µg/mL. In particular, PEFE-AuNPs significantly reduced the number of colonies compared to PEFE at the same concentrations. Moreover, 100 µg/mL of PEFE-AuNPs resulted in a greater inhibition of colony formation than the positive control (50 µM of cisplatin), suggesting the potential application of PEFE-AuNPs in cancer treatment. Taken together, our results suggest that the anticancer activity of PEFE-AuNPs involves the inhibition of colony formation without inducing significant toxic effects on normal cells.

### 3.3. Mitochondrial Damage Is Induced by PEFE-AuNPs in AGS Cells

**Figure 4:**
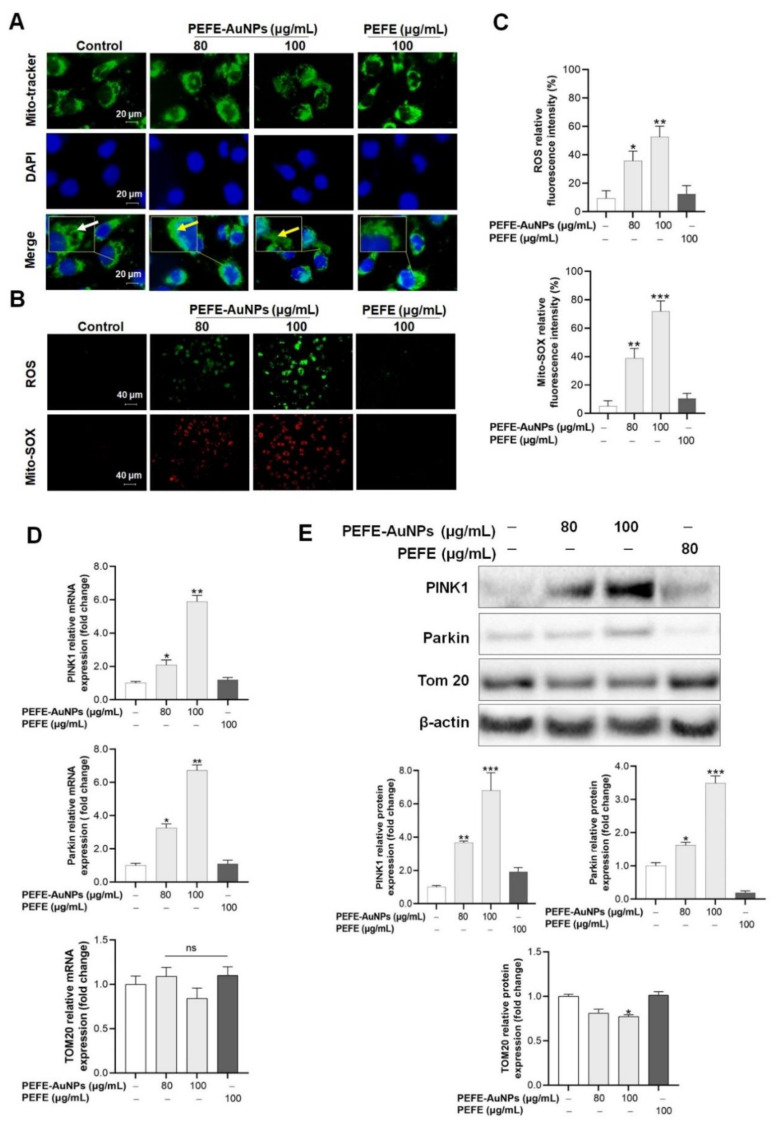
(**A**) Fluorescence microscopic images of mitochondrial morphology of AGS cells stained with Mito-tracker. The white and yellow arrows indicate healthy and damaged mitochondria, respectively. (**B**,**C**) Production of mitochondrial reactive oxygen species (ROS) and fluorescence microscopic images of mitochondrial morphology of AGS cells stained with Mito-SOX. (**D**,**E**) Effect of PEFE-AuNPs and PEFE on the expression of apoptosis-related mRNA and proteins. The western blotting images were quantified by Image J software. The asterisks on the column indicate significant differences between each tested sample and untreated control. *, *p* < 0.05, **, *p* < 0.01, ***, *p* < 0.001. ns, not significant.

We investigated the molecular mechanism of the anticancer activity of PEFE-AuNPs against AGS cells. It is well known that mitochondrial damage is an important feature in the early stages of programmed cell death [40]. In particular, during caspase-induced apoptosis, significant mitochondrial dysfunction is caused by the loss of mitochondrial membrane potential (Δψm) and the production of ROS [41]. In the present study, we investigated the mitochondrial morphology in AGS cells treated with PEFE-AuNPs through fluorescence microscopy following staining with the mitochondria-specific probe Mito-tracker (Figure 4A). The results showed that the AGS cells treated with PEFE-AuNPs had fragmented mitochondria, whereas those in the control group and those treated with PEFE had normal tubular mitochondria. Fragmented mitochondria commonly have a round, punctate morphology, suggesting the occurrence of mitochondrial fission or fragmentation [42] Since high concentrations of mitochondrial superoxide can induce intracellular oxidative damage and subsequent apoptosis of cancer cells [42,43], we evaluated the ROS production in AGS cells via MitoSOX assay (Figure 4B). A dose-dependent production of Mito-SOX and ROS was observed in the cells treated with PEFE-AuNPs but not in those treated with PEFE, suggesting that excessive ROS accumulation in the presence of PEFE-AuNPs may induce apoptosis in AGS cells. The results of the ROS and Mito-SOX production were quantified for statistical analysis (Figure 4C).

We then investigated the molecular mechanism underlying the apoptosis in AGS cells treated with PEFE-AuNPs. Previous studies have shown that the Parkin-mediated pathway involving PINK1 is one of the main pathways in anticancer therapy [44]. As the Δψm is lost, PINK1 transiently accumulates on the outer membrane of mitochondria, and Parkin is selectively recruited and forms a large complex that is integrated into the dysfunctional mitochondria to induce autophagy [45]. In this study, the mRNA expression and protein levels of PINK1 and Parkin in AGS cells treated with PEFE-AuNPs significantly increased in a dose-dependent manner compared to those in the control group or PEFE-treated cells (Figure 4D,E). TOM20 is a central component of the translocase of outer membrane (TOM) family, which is responsible for protein transport into the mitochondrial matrix [46]. PEFE-AuNPs at a concentration of 100 μg/mL significantly reduced the expression level of TOM20 compared with the negative control. Our results suggest that the mitochondrial disruption induced by PEFE-AuNPs is associated with the PINK1-Parkin-mediated Δψm reduction, resulting in the induction of apoptosis in AGS cells.

### 3.4. Apoptosis Is Induced by PEFE-AuNPs in AGS Cells

**Figure 5:**
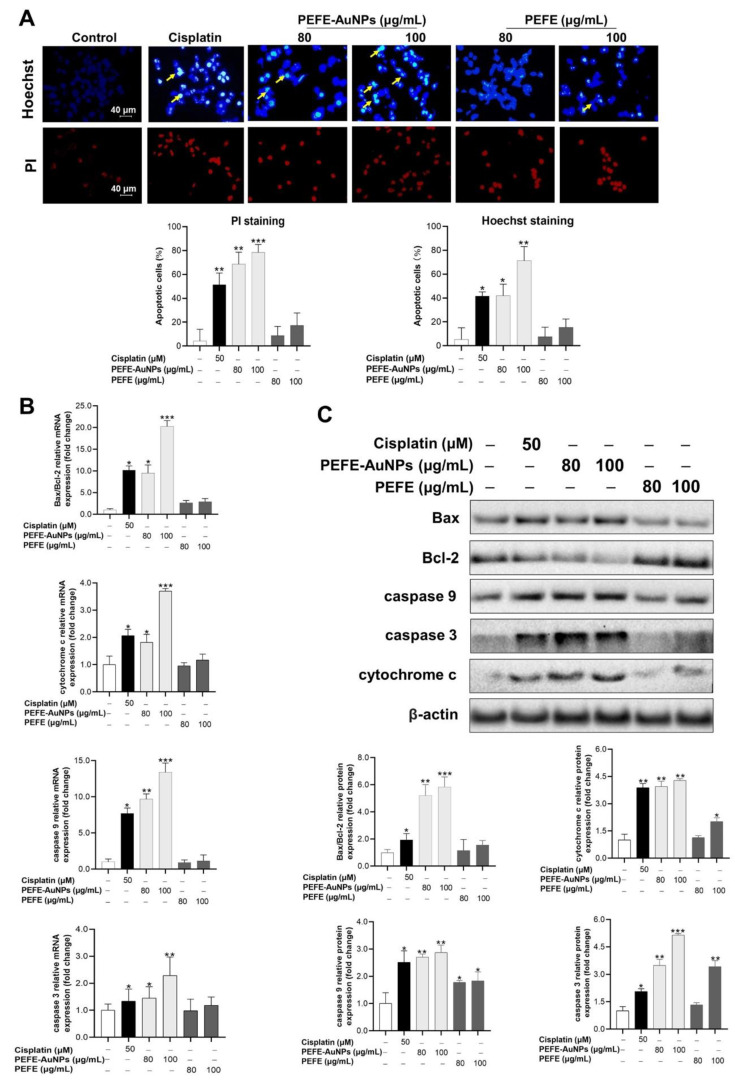
(**A**) Fluorescence microscopic images of AGS cells stained with Hoechst and propidium iodide (PI). The yellow arrows in the pictures indicate fragmented nuclei with condensed chromatin, and results were quantified using the Image J software. (**B**) Effect of PEFE-AuNPs and PEFE on the expressions of apoptosis-related genes in AGS cells. The cisplatin was used as positive control. (**C**) The expression level of each protein was determined by western blotting, and images were quantified by Image J software. The asterisks on the column indicate significant differences between each tested sample and untreated control. *, *p* < 0.05, **, *p* < 0.01, ***, *p* < 0.001.

Apoptosis, an important process in homeostasis, destroys abnormal cells, including cancer cells. Some strategies for cancer therapy involve the inhibition of tumor cell replication via apoptosis [47,48]. Fluorescence microscopy images of Hoechst- and PI-stained AGS cells treated with cisplatin, PEFE-AuNPs, and PEFE are shown in Figure 5A. Hoechst staining revealed that indistinct blue nuclei were present in the control group, indicating the absence of apoptosis. In contrast, significant pathological alterations were observed in the cells treated with PEFE-AuNPs (80 and 100 μg/mL), indicating the occurrence of apoptosis. Moreover, a greater magnitude of apoptosis was induced in the cells treated with PEFE-AuNPs than in those treated with PEFE. PI staining, in which only dead cells are stained red, revealed that PEFE-AuNPs significantly increased the number of apoptotic cells in a dose-dependent manner compared to PEFE.

We then investigated the molecular mechanism underlying the apoptosis induced by PEFE-AuNPs. Of the various apoptotic pathways, the Bax/Bcl-2 signaling pathway plays the main role in the activation of the mitochondrial apoptotic pathway, which enhances the permeability of the mitochondrial membrane and the release of cytochrome c, followed by the activation of caspases (caspases 3 and 9) and subsequent induction of apoptosis [49,50]. The expression levels of apoptosis-related proteins, including Bax, Bcl-2, cytochrome c, caspase 9, and caspase 3, are shown in Figure 5B. PEFE-AuNPs significantly increased the expression of all the assessed mRNAs in AGS cells in a dose-dependent manner compared to the negative control and PEFE. These results were consistent with the results of the intracellular protein expression analysis by western blotting (Figure 5C), which revealed that PEFE-AuNPs significantly increased the levels of apoptosis-associated proteins, such as Bax/Bcl-2, cytochrome c, caspase 9, and caspase 3. These results suggest that apoptosis in the treated AGS cells is likely caused by PEFE-AuNPs, which activate intracellular signaling pathways through Bax/Bcl-2 and caspase 9/caspase 3.

### 3.5. PEFE-AuNPs Promote Apoptosis by Inhibiting Autophagy in AGS Cells

**Figure 6:**
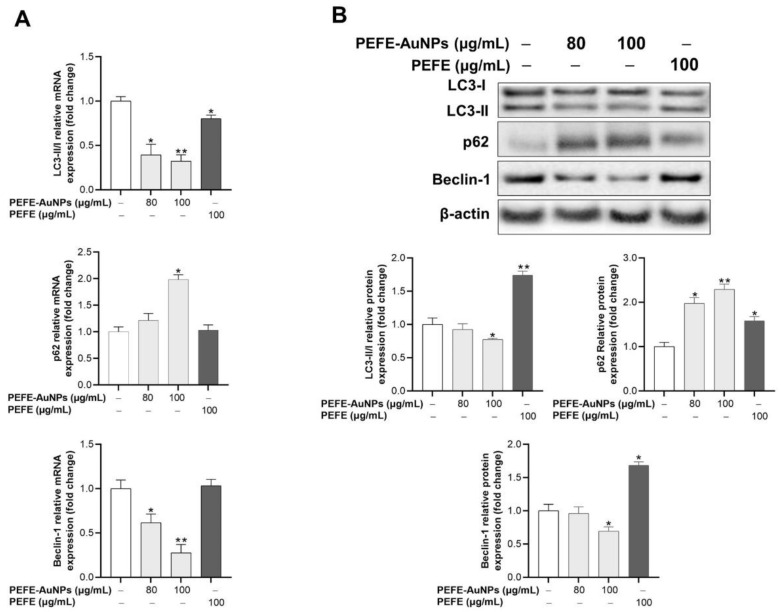
The effect of PEFE-AuNPs and PEFE on the expression of autophagy-related (**A**) mRNA and (**B**) proteins in AGS cells. The western blotting images were quantified by Image J software. The asterisks on the column indicate significant differences between each tested sample and untreated control. *, *p* < 0.05, **, *p* < 0.01.

It is well known that apoptosis is generally suppressed by autophagy, and autophagy is blocked by apoptosis-associated caspase activation [50]. Although both self-destructive processes sequentially occur in the same cell (autophagy generally precedes apoptosis), they act by cross-regulating each other in an inhibitory manner [50]. Thus, we investigated whether the anticancer activity of PEFE-AuNPs is associated with autophagy. LC3, sequestosome 1/p62 (p62), and Beclin-1 have been determined as markers for monitoring autophagy and autophagy-related processes [51,52]. LC3, a model substrate to measure autophagic flux, is structurally transformed through lipid modification and subsequent conjugation with phosphatidylethanolamine to form the membrane-bound LC3-II, which can be detected by immunoblotting or immunofluorescence analysis [53]. p62 is another widely used marker to monitor autophagic activity, because it directly binds to LC3 and is selectively degraded by autophagy [54,55]. Beclin-1 is also a marker for autophagy, as the caspase-mediated breakdown of Beclin-1 in apoptosis affects autophagy inhibition [56]. As shown in Figure 6A, PEFE-AuNPs was associated with a significant downregulation of the mRNA expression of LC3-II/LC3-I and Beclin-1 and a dose-dependent upregulation of the mRNA expression of p62. These results were consistent with the results of the intracellular protein expression analysis by Western blotting (Figure 6B).

**Figure 7:**
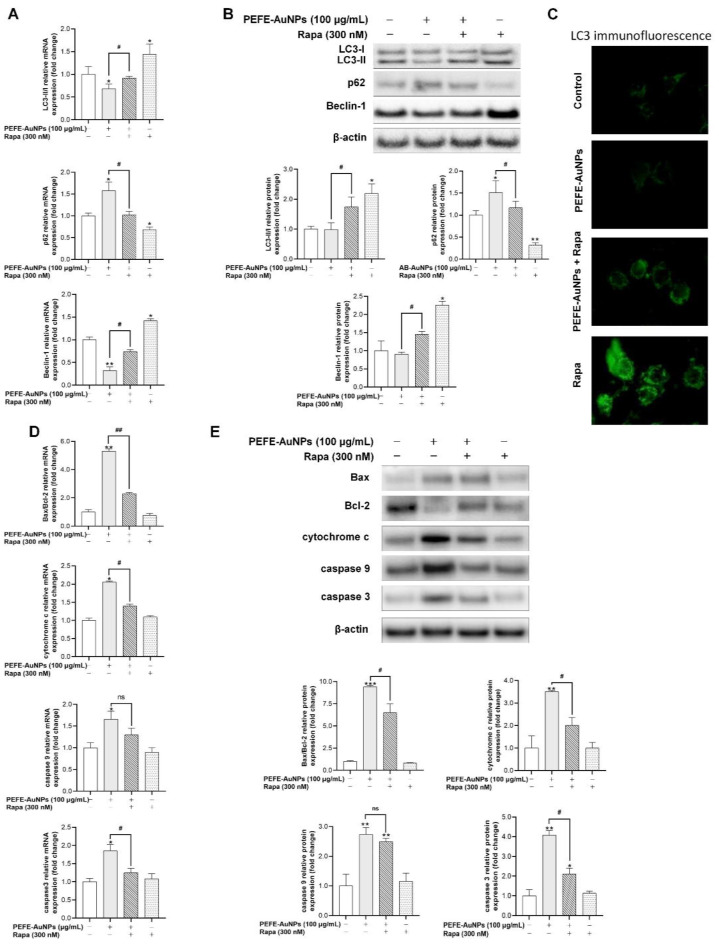
The effect of PEFE-AuNPs and PEFE on the expression of autophagy-related (**A**) mRNA and (**B**) proteins in autophagy-stimulated AGS cells treated with Rapamycin (Rapa). (**C**) Fluorescence microscopic images in Rapa-treated AGS cells. The effect of PEFE-AuNPs and PEFE on the expression of apoptosis-related (**D**) mRNA and (**E**) proteins in autophagy-stimulated AGS cells treated with Rapamycin (Rapa). The western blotting images were quantified by Image J software. The asterisks on the column indicate significant differences between each tested sample and untreated control, and the crosshatch patterns indicate significant differences between PEFE-AuNPs and Rapa. * and #, *p* < 0.05, ** and ##, *p* < 0.01. ***, *p* < 0.001. ns, not significant.

On the contrary, the autophagy inducer rapamycin (Rapa), which can strengthen autophagy-related mechanisms, was used to explore the detailed mechanism of action of the cytotoxicity of PEFE-AuNPs against AGS cells. qRT-PCR analysis and western blot analysis revealed that the expression of LC3-II/LC3-I and Beclin-1 was significantly upregulated in Rapa-treated AGS cells but downregulated in cells treated with PEFE-AuNPs and Rapa (Figure 7A,B). In contrast, the expression of p62 in cells treated with PEFE-AuNPs and Rapa was increased compared to that in cells treated with Rapa alone. Fluorescence microscopy results showed that PEFE-AuNPs inhibited LC3 expression in Rapa-treated AGS cells (Figure 7C). Finally, the expression levels of apoptosis-related signaling molecules, such as Bax, Bcl-2, cytochrome c, caspase 9, and caspase 3, were determined in AGS cells with Rapa-induced autophagy. PEFE-AuNPs significantly upregulated the expression of these molecules in a dose-dependent manner, indicating that Rapa-stimulated autophagy was significantly inhibited by PEFE-AuNPs in AGS cells (Figure 7D,E). Thus, our results suggest that PEFE-AuNPs can act as an anticancer agent by facilitating apoptosis and inhibiting autophagy in AGS cells.

## 4. Conclusions

In the present study, PEFE-AuNPs with particle sizes of 5–60 nm were prepared with PEFE and HAuCl_4_·3H_2_O via microbial biosynthesis using *B. lactis*. The optimized conditions for the biosynthesis were determined. Through various analytical methods, such as UV-Vis spectrometry, FE-TEM, EDX, elemental mapping, XRD, SAED, and FT-IR spectroscopy, the synthesized PEFE-AuNPs were determined to have a more favorable thermal stability than PEFE and were shown to be intracellularly absorbed by AGS cells. Compared to PEFE, PEFE-AuNPs showed an increased cytotoxicity against AGS cells but not against normal cells, suggesting that PEFE-AuNPs exert a selective toxic effect on cancer cells. Through molecular mechanism studies, we demonstrated that the cytotoxic effect of PEFE-AuNPs was associated with the activation of the cellular apoptosis pathway, which includes Bax/Bcl-2, and caspase 9/caspase 3, and is associated with excessive mitochondrial ROS production. Moreover, autophagy was determined to be inhibited in the presence of PEFE-AuNPs. As such, this study is the first to show that PEFE-AuNPs may serve as a novel therapeutic agent for treating GC. Furthermore, our study provides basic data for developing novel anticancer candidates and understanding their anticancer mechanisms. Furthermore, our results are a good starting point for the development of new anticancer products based on PEFE-AuNPs.

## Data Availability

Data are contained within the article.

## Supplementary Materials

The following are available online at https://www.mdpi.com/article/10.3390/nano11051260/s1, Figure S1: Reaction conditions-dependent evolution of UV–vis spectra of synthesized nanoparticles to maximize the yield of nanoparticles, Figure S2: Stability test of PEFE-AuNPs depending on storage period. Table S1: Real-time PCR primers used in the qRT-PCR assays.
