# Supplementary material for: Gold Nanoparticles Prepared with Phyllanthus emblica Fruit Extract and Bifidobacterium animalis subsp. lactis Can Induce Apoptosis via Mitochondrial Impairment with Inhibition of Autophagy in the Human Gastric Carcinoma Cell Line AGS
Source: Nanomaterials (Basel). 2021 May 11;11(5):1260. doi: 10.3390/nano11051260 (PMC8150816; doi:10.3390/nano11051260)
Supplement: Supplementary file 1 [file nanomaterials-11-01260-s001.zip › nanomaterials-1202994-supplementary.pdf]

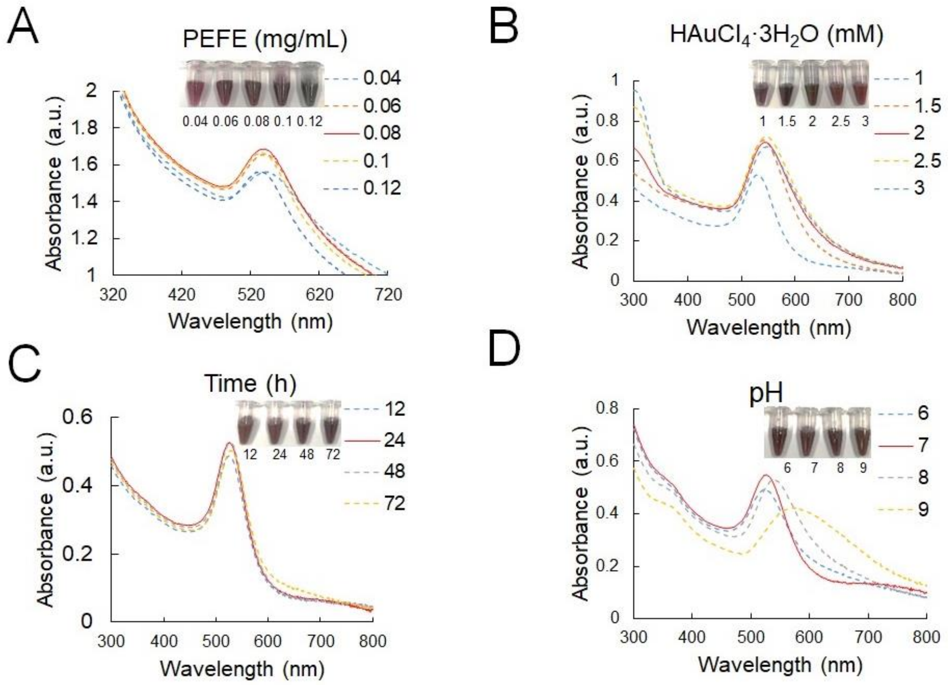

Supplementary Figure S1. Reaction conditions-dependent evolution of UV–vis spectra of synthesized nanoparticles to maximize the yield of nanoparticles. (**A**) Concentration of *Phyllanthus emblica* extract (*P. emblica*); (**B**) concentration of $HAuCl_4 \cdot 3H_2O$; (**C**) reaction time and (**D**) reaction pH evolution of UV–vis spectra of *Phyllanthus emblica* gold nanoparticles (PEFE-AuNPs) and the respective photograph which shows the color change pattern during nanoparticle synthesis of PEFE-AuNPs.

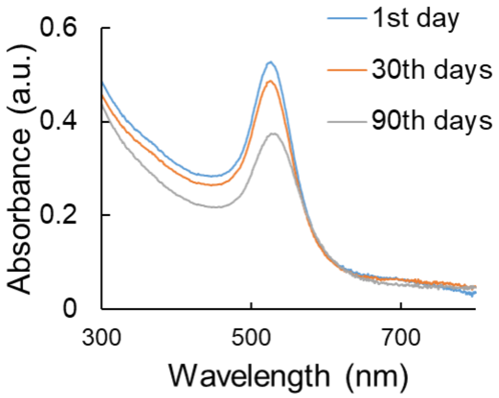

Supplementary Figure S2. Stability test of PEFE-AuNPs depending on storage period.

Table S1. Real-time PCR primers used in the qRT-PCR assays

Target gene	Forward Primer sequence (5′-3′)	Reverse Primer sequence (5′-3′)
PINK1	TCCTCCAGCGAAGCCATCTTAAGC;	TGCAGCACATTTGCAGCTAAGCGT;
Parkin	TTCATCTACTGCAAAGGCCCCTGC;	TCCCATTTGCAGCACGCATTCCTC;
TOM20	TTCTGACCAAGCTTCCGACCATTA	ACTGACCTAATGCTGAGATGGAAC
Bax	AGCAAACTGGTGCTCAAGGC;	CCACAAAGATGGTCACTGTC;
Bcl-2	GTGGTGGAGGAACTCTTCAG;	GTTCCACAAAGGCATCCCAG;
Cytochrome c	GAGGCAAGCATAAGACTGG;	TACTCCATCAGGGTATCCTC;
Caspase 9	AGTTCCCGGGTGCTGTCTA;	GCCATGGTCTTTCTGCTCA;
Caspase 3	CCTCAGAGAGAGACATTCATG;	GCAGTAGTCGCCTCTGAAG;
LC3-I	ACATGAGCGAGTTGGTCAAGATCA;	GATGGATTCTGGCCCAGTCATATT;
LC3-II	ATAATTAGAAGGCGCTTACAGCTC;	TGGCAGGTTCTCTTCTCTAGATCT;
Beclin-1	GCCAGGATGGTGTCTCTCGAAGAT;	GTGGAAGGTGGCATTGAAGACATT;
SQSTM1(P62)	ACCTGTCTGAGGGCTTCTCGCACA;	CTCTTCTCCTCTGTGCTGGAACTC;
